# Analysis of tall fescue ESTs representing different abiotic stresses, tissue types and developmental stages

**DOI:** 10.1186/1471-2229-8-27

**Published:** 2008-03-04

**Authors:** MA Rouf Mian, Yan Zhang, Zeng-Yu Wang, Ji-Yi Zhang, Xiaofei Cheng, Lei Chen, Konstantin Chekhovskiy, Xinbin Dai, Chunhong Mao, Foo Cheung, Xuechun Zhao, Ji He, Angela D Scott, Christopher D Town, Gregory D May

**Affiliations:** 1 Forage Improvement Division, The Samuel Roberts Noble Foundation, 2510 Sam Noble Parkway, Ardmore, OK 73402, USA; 2 Plant Biology Division, The Samuel Roberts Noble Foundation, 2510 Sam Noble Parkway, Ardmore, OK 73402, USA; 3 Virginia Bioinformatics Institute, 1750 Kraft Drive Suite 1400, Virginia Tech, Blacksburg, VA 24061, USA; 4 The J. Craig Venter Institute, 9712 Medical Center Drive, Rockville, MD 20850, USA; 5 USDA-ARS, The Ohio State University & OARDC, 1680 Madison Avenue, Wooster, OH 44691, USA; 6 National Center for Genome Resources, 2935 Rodeo Park Drive East, Santa Fe, NM 87505, USA

## Abstract

**Background:**

Tall fescue (*Festuca arundinacea *Schreb) is a major cool season forage and turf grass species grown in the temperate regions of the world. In this paper we report the generation of a tall fescue expressed sequence tag (EST) database developed from nine cDNA libraries representing tissues from different plant organs, developmental stages, and abiotic stress factors. The results of inter-library and library-specific *in silico *expression analyses of these ESTs are also reported.

**Results:**

A total of 41,516 ESTs were generated from nine cDNA libraries of tall fescue representing tissues from different plant organs, developmental stages, and abiotic stress conditions. The *Festuca *Gene Index (FaGI) has been established. To date, this represents the first publicly available tall fescue EST database. *In silico *gene expression studies using these ESTs were performed to understand stress responses in tall fescue. A large number of ESTs of known stress response gene were identified from stressed tissue libraries. These ESTs represent gene homologues of heat-shock and oxidative stress proteins, and various transcription factor protein families. Highly expressed ESTs representing genes of unknown functions were also identified in the stressed tissue libraries.

**Conclusion:**

FaGI provides a useful resource for genomics studies of tall fescue and other closely related forage and turf grass species. Comparative genomic analyses between tall fescue and other grass species, including ryegrasses (*Lolium *sp.), meadow fescue (*F. pratensis*) and tetraploid fescue (*F. arundinacea var glaucescens*) will benefit from this database. These ESTs are an excellent resource for the development of simple sequence repeat (SSR) and single nucleotide polymorphism (SNP) PCR-based molecular markers.

## Background

On a worldwide basis, grasslands occupy twice the land area of grain crops [1]. Tall fescue (*Festuca arundinacea *Schreb) is a major cool season forage and turf grass with a genome size of approximately 6 × 10^3 ^Mbp and an out-crossing mode of reproduction [2]. Tall fescue is a hexaploid species that contains three genomes (P, G1, and G2). The P (2x) genome originates from *F. pratensis *while the G1 and G2 (4x) genomes are derived from *F. arundinacea *var 'Glaucescens' [3]. Tall fescue is closely related to a number of *Lolium *species including perennial ryegrass (*Lolium perenne*) and annual ryegrass (*Lolium multiflorum*). The *Festuca*-*Lolium *complex contains well-adapted, highly productive grass species that are widely distributed in many parts of the world [4]. These cultivated forage grasses provide numerous benefits to humans, including providing feed and fodder for millions of dairy and beef cattle, horses, sheep, and countless wild animals [5]. Turf grass production for use in golf courses and lawns is a multi-billion dollar U.S. industry. Besides the direct economic benefits gained from forage and turf grasses, their contributions in soil conservation, environmental protection, recreation, and aesthetics are substantial.

To date, complete genome sequences are available for only two plant species, *Arabidopsis thaliana *and *Oryza sativa*, both with relatively small genome sizes compared to most crop plants. The genome of tall fescue is approximately 14 times larger than that of rice. It is unlikely that a complete genome sequence will be available for tall fescue or any other forage or turf grass species in the near future. For grass species with large genomes, focused large-scale development and analysis of ESTs can provide a basis for gene discovery and the determination of gene function [6].

The out-crossing nature of reproduction and genome complexity of tall fescue make conventional molecular studies difficult and inefficient. Thus molecular studies in tall fescue have lagged far behind those of major cereal species. Tall fescue EST and database resources will be useful for comparative genomic analyses of this important plant species with other major grass species, including rice [7], and help cross-species transfer of genetic knowledge from the well characterized species (e.g., rice) to those less studied.

Here we report the generation of 41,516 tall fescue ESTs characterized from nine cDNA libraries representing tissues from different plant organs, developmental stages, and abiotic stress factors. We also report the results of inter-library and library-specific *in silico *EST expression analyses.

## Results and discussion

### Festuca cDNA libraries and generation of unigene sets

More than 49,000 EST sequences were generated from nine tall fescue cDNA libraries constructed from tissues representing various tissue types (leaves, roots, stems, and floral meristems), growth stages (young seedlings, juvenile vegetative stage, and early reproductive stages), and abiotic stress factors (drought, heat, and multi-factor field stress) (Table 1). DNA sequencing success rates varied between 75 – 97% for all libraries with an overall average length of 536 bp. The young seedling (SD1) and heat stressed shoot (HSS) libraries had the highest (598 bp) and lowest (505 bp) average trimmed EST lengths, respectively (Table 1). Sequences less than 50 nucleotides in length (7.7%), low quality sequences (1.8%), chimeric sequences (1.8%), and contaminated sequences (3.4%) were removed from the data set. A total of 41,834 EST sequences were deposited in the GenBank dbEST.

**Table 1:** Tall fescue cDNA library and ESTs summary

**cDNA library name**^a^	**NCBI dbEST accession No.**	**Average length (bp)**	**No. of ESTs**	**No. of TC**^c^	**No. of singleton**	**No. of unique TC**^e^	**No. of ESTs in unique TC**	**No. of unique sequences**^f^
Drought stressed root (DR1)	DT674223 to DT679214	518	4,954 (81)^b^	613	191 (3.9)^d^	170	955 (19.3)^d^	361 (2.0) ^g^
Drought stressed shoot (DS1)	DT679215 to DT684265	508	4,972 (75)	1621	1,704 (34.3)	135	305 (6.1)	1,839 (10.3)
Field stressed shoot (FSS)	DT684266 to DT685476	579	1,210 (97)	367	741 (61.2)	14	32 (2.6)	755 (4.2)
Floral meristem (TFM)	DT685477 to DT690490	522	4,965 (76)	1624	1,263 (25.4)	519	1376 (27.7)	1,782 (10.0)
Heat stressed shoot (HSS)	DT690491 to DT695609	505	5,090 (82)	1743	1,551 (30.5)	506	1202 (23.6)	2,057 (11.6)
Greenhouse grown leaf (LF1)	DT695610 to DT700548	518	4,885 (89)	1346	1,079 (22.1)	158	383 (7.8)	1,237 (6.9)
Greenhouse grown root (RT1)	DT700549 to DT706237	554	5,679 (92)	1917	1,691 (29.8)	274	661 (11.6)	1,965 (11.0)
Field grown stem (ST1)	DT706238 to DT711272	554	4,991 (87)	1627	1,866 (37.4)	194	447 (9.0)	2,060 (11.6)
Young seedling (SD1)	DT711273 to DT716056	598	4,770 (96)	1643	1,831 (38.4)	179	401 (8.4)	2,010 (11.3)

**Total**		**536**	**41,516**					

^a^Details on plant growing conditions, tissue sampling, and library construction for each cDNA library are available at NCBI.

^b^Number within parenthesis indicates percent sequencing success rate.

^c^Number of tentative consensus (TC) sequences generated from EST library members.

^d^Percentage (%)of the total number of ESTs in each library.

^e^Number of unique TCs assembled from ESTs only present in each library.

^f^Number of unique sequences only present in each library, including singleton and unique TC.

^g ^Fraction of unique sequences in this library to all 17,806 unique sequences.

**Figure 1:**
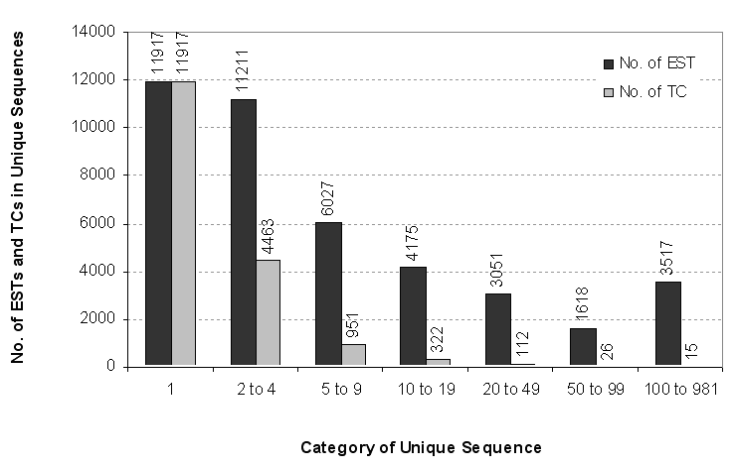
**Distribution of *Festuca *unique sequences**. Numbers on bar tops indicate the number of ESTs and TCs for each category.

In collaboration with The Institute for Genomic Research (TIGR) the *Festuca *Gene Index (FaGI) containing 41,516 high-quality ESTs was established [8]. A library-based breakdown of the ESTs is shown in Table 1. Cluster analysis revealed 17,806 unigene sequences that included 11,917 singletons and 5,889 tentative consensus (TC) sequences assembled from 29,599 ESTs (Table 2). More than 67% of the unique sequences or 29% of all ESTs were singletons. The number of ESTs in the TCs ranged from two to 981 ESTs with an average of five ESTs per TC (Figure 1). More than 99% of TCs are less than 2 kb in length, including TCs <1 kb (78%) and those between 1 to 2 kb (21%) (data not shown). The longest tentative consensus (TC 2128) was 3,215 bp and encodes a rice beta-galactosidase homologue (Table 2). Approximately 30% of unique sequences are expressed at a low to medium level, i.e. they are represented by TCs assembled from two to nine ESTs and accounted for 42% of all sequences. Only 2.7% of unique sequences are highly expressed as they are represented by TCs comprising more than ten ESTs. The highly expressed genes covered 30% of the ESTs (Figure 1). Among them were 15 TCs that consist of more than 234 ESTs each. Eight of these highly expressed transcripts, derived from stem or leaf tissue libraries, demonstrate sequence similarities to genes with known function, most of which are involved in carbon fixation (rubisco, photorespiration, photosystem II) and carbon metabolism. TC1995, comprising the largest number of ESTs (981), is most similar to a rice hypothetical protein of unknown function.

**Table 2:** Tall fescue ESTs summary statistics on clustering analyses

	**Number of sequence**	**Minimum length (bp)**	**Maximum length (bp)**	**Average length (bp)**
**TCs**	5,889	106	3,215	810
**ESTs which appear in TCs**	29,599	100	736	537
**Singletons**	11,917	100	731	532
**Unique sequences**^a^	17,806	100	3,215	624
**All EST**	41,516	100	736	536

^a ^Unique sequences = TCs + singletons

To compare ESTs characterized from each library, the numbers of singletons, ESTs in unique TCs and unique ESTs were calculated. Singleton percentages were calculated by dividing singletons by the total number of ESTs in each library. Singleton percentages ranged from 3.9% to 61% in the DR1 and FSS libraries, respectively. Only 2.6% of ESTs assembled into unique TCs in the FSS library. The percentage of unique ESTs, which include both singleton and ESTs in unique TCs, is based on ESTs present in each library, and therefore, suggests library specificity of ESTs. Almost two-thirds of the ESTs from the FSS library are unique to this library (Table 1).

### Annotation of the unigene sets

BLASTX [9] was conducted against the GenBank non-redundant protein (nr) database to assign putative identity to the *Festuca *unigene set. Based on an E-value cutoff of ≤ 1 e^-5^, 68% (12,077) of the *Festuca *unigenes showed significant levels of similarity to nr. Approximately 28% (3,410) of these protein homologues were annotated as unknown, hypothetical, or expressed proteins, while the remaining (8,667) correspond to proteins with putative known functions.

**Figure 2:**
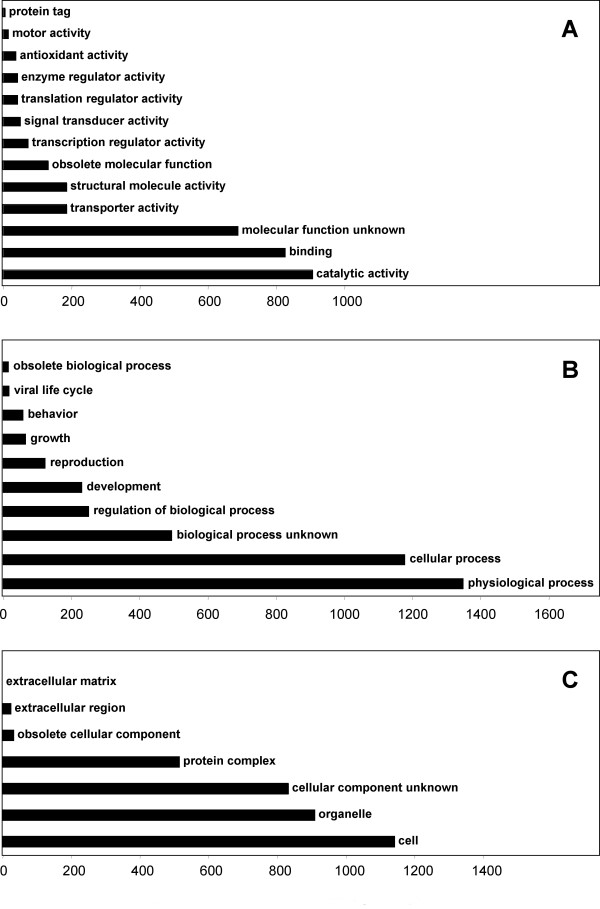
**Distribution of *Festuca *unigenes with putative functions assigned through Gene Ontology annotation**. A, Molecular function. B, Biological process. C, Cellular component. Assignments are based on the data available at FaGI (1.0).

Functional annotation was assigned by mapping unigenes onto the Gene Ontology Consortium [10] structure using the FaGI (1.0). Unigenes with assigned putative functions were classified into three ontologies: molecular function, biological process, and cellular component by controlled GO vocabulary [10]. In total, 2,410 unigenes (including 1,762 TCs and 648 singletons) were mapped to one or more ontologies, with multiple assignments possible for a given protein within a single ontology. Thus, 2,305 assignments were made to the molecular function ontology, with more than 75% of these in the catalytic activity and binding category annotations such ligase, transferase, helicase, and nucleotide binding proteins (Figure 2A). Branch child terms for transporter and transcription regulator activities revealed several genes implicated in water channel (e.g., Q40047 and Q8S4X5), carbohydrate transporter (e.g., Q8GTR0 and Q5XF02), as well as predicted transcription factors with putative roles in stress responses (e.g. heat shock factor RHSF6, HMG1/2-like protein, MYB-like protein Q4L214 and Q4JL76, and zinc finger protein genes Q5Z9H7 and O82115). Under the biological process ontology, the majority of the 1,773 assignments were to the physiological process (76%) and cellular process (66%) categories, with frequent sub-classification into the response to stress, response to external stimulus, and cell growth and maintenance categories (Figure 2B). The abundance of stress-related annotations is not surprising, considering that a significant portion of the ESTs were generated from tissues subjected to abiotic stresses. Of the 1,725 unigenes mapped into cellular component ontologies, the largest groups were assigned into the cell (66%) and organelle (53%) categories (Figure 2C).

### In silico analysis of gene expression

**Figure 3:**
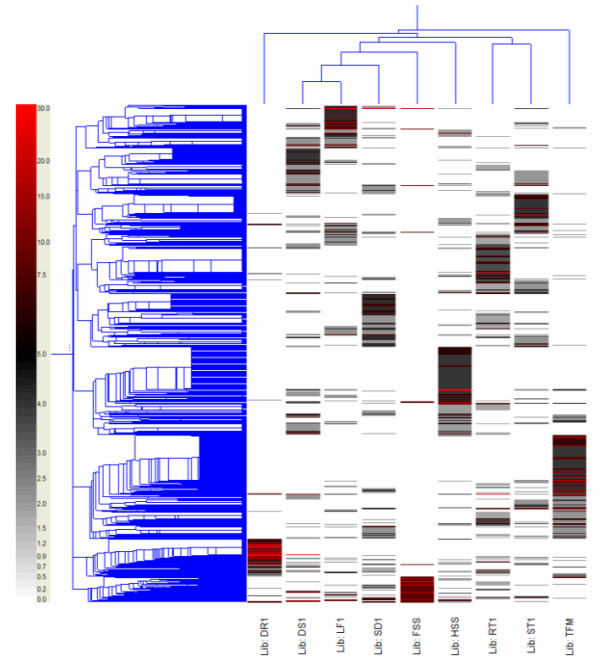
**Clusters of 5,889 tentative consensus (TC) sequences exhibiting differential EST abundance in organ/stress-specific cDNA libraries**. Columns represent the nine tall fescue cDNA libraries. DS1: Drought stressed shoot, LF1: Greenhouse grown leaf, SD1: Young seedling, FSS: Field stressed shoot, HSS: Heat stressed shoot, TFM: Floral meristem, RT1: Greenhouse grown root, ST1: Field grown stem, DR1: Drought stressed root. The number of ESTs forming a TC in each cDNA library is presented in coloured rows. The correlation map resulting from clustering of the TCs is given at the left. The dendrogram on top illustrates the relationship between the cDNA libraries/plant organs analyzed. Nine clusters (A to I) are indicated on the right, with number of TCs included in each cluster.

To identify putative differentially expressed genes, *in silico *expression analysis were conducted by hierarchical clustering [11] of expression levels of all 5,889 TCs in the nine cDNA libraries, represented as EST counts normalized according to library size, using GeneSpring 7.2. The libraries were separated into four arbitrary groups based on clustering analysis (Figure 3). Five libraries, including DS1, LF1, SD1, FSS, and HSS formed the largest group by step further relationships. Three libraries (DS1, FSS, and HSS) were from stressed above ground tissues, and therefore, may share similar gene expression memberships. Another group contains RT1 and ST1 libraries, which contain apical meristem tissues active in cell division, elongation, and differentiation. Expression of ESTs generated from stressed root tissue (DR1) significantly differed from other libraries, and was therefore grouped into a third block. As expected, TFM (floral meristem) library, representing ESTs from reproductive tissue showed unique expression that differentiated from all other tissue type libraries.

Genes sharing quantitatively and functionally related expression patterns were also identified. Based on their library specificity, TCs were classified into nine major clusters (A to I) as shown in Figure 3. Each library was assigned a major cluster containing genes specifically expressed in the particular tissue type or stress treatment of the library. Number of TCs included in each cluster was from 303 (cluster I) to 1,232 (cluster G) (Figure 3).

The majority of the genes in cluster A were generated from leaf tissue-derived ESTs. Not surprisingly, numerous photosynthesis and carbon fixation genes were highly expressed, e.g., protein homologue of chloroplast carbonic anhydrase and rubisco activase gene transcripts were represented by more than 570 and 244 ESTs, respectively from this library. In addition, more than 40 TCs in cluster A were the homologues of chlorophyll a/b binding (CAB) and photosystem II (PSII) proteins. Abundant stem tissue ESTs were grouped in cluster C. Included in this cluster are homologues of enzymes involved in cytosolic glycolysis. For example, ESTs of genes that encode for sucrose synthase, glucose dehydrogenase, malate dehydrogenase, and fructose 1,6-bisphosphate aldolase were present at 20 or greater copies in the stem library.

Cluster D includes genes specifically expressed in roots (RT1) and contains high-copy number ESTs encoding different classes of methionine synthases and methyl transferases. ESTs of a barley metallothionein-like protein (MT) homologue were highly expressed in the root (RT1) library and were clustered into six TCs comprised of 139 copies. MT1, one of the four classes of metallothioneins was previously reported to express significantly higher in root than in other tissues such as leaf and flower [12].

The genes associated with clusters E and G are mainly expressed in actively dividing (young seedling and floral meristem) tissues. Products of genes in these clusters are necessary for cell cycle regulation, transcription and translation. Germination-specific cluster E contains a number of genes coding for transcriptional factors (e.g., shaggy-related protein kinase, CBL-interacting protein kinase, MYB29 protein). In addition, genes involved in photosynthesis were also highly expressed in young seedlings. G, the largest cluster comprised of 101 TCs, contains significant numbers of floral meristem ribosomal and histone protein isoforms that comprise approximately 8% of the members of this cluster. Histone H2, a meristem-specific gene homologue, was present in 19 TCs from this library. Histone H2 mRNA is transiently accumulated during a period of the cell cycle that mostly overlaps the S phase [13]. Our results show increased expression of these transcripts which may be indicative of active cell division in floral meristem tissues [14].

Cluster B, F, H, and I represent drought-stressed (DS1), heat-stressed shoot (HSS), drought stressed root (DR1), and field stressed shoot (FSS) libraries, respectively. A total of 2,338 TCs were contained in these four clusters, and accounted for 40% of all contigs. Large numbers of stress response genes were found in these four clusters, including homologues of heat-shock and oxidative-stress proteins, and various classes of transcription factors. Differences in stress-related gene expression were also observed among libraries. Perhaps this is due to differences in stress mechanisms and in other environmental and biological factors among libraries. For example, 23 TCs coding for different classes of heat-shock proteins (cluster F) were found in the heat-stressed shoot library. This is significantly higher than the number of heat-shock TCs observed in other stress libraries. Heat stress induced by high temperatures can result in damage to the photosynthetic apparatus [15] thus many chloroplast and photosynthesis related genes found in this cluster were expected. TC1995 had high EST numbers in both drought-stressed shoot (107) and drought-stressed root (1,873) libraries, but was not detected in other libraries (non-stressed shoot or root, other tissue types, or stress treatments). This novel drought-specific TC has no similarity to any gene in the nr database. Another highly expressed TC, a homologue of a DNA binding protein, contained 1,272 ESTs in drought-stressed shoot and 56 EST in drought-stressed root libraries.

### Environmental conditions and gene expression

**Figure 4:**
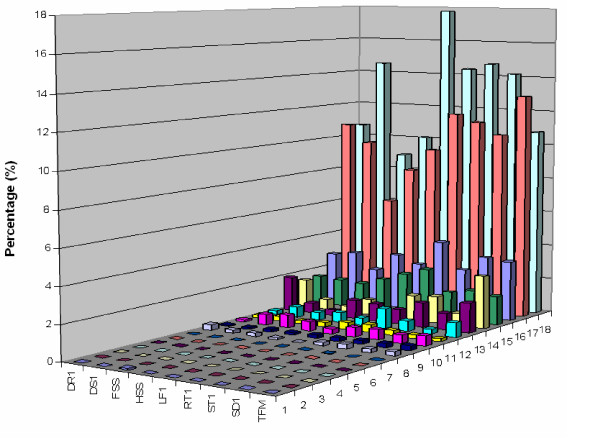
**Library comparisons based on gene ontology Molecular Function assignments**. X axis: library (as listed in Table 1). Y axis, GO function: 1. chaperone regulator activity, 2. triplet codon-amino acid adaptor activity, 3. protein tag, 4. energy transducer activity, 5. chemoattractant activity, 6. chemorepellant activity, 7. nutrient reservoir activity, 8. motor activity, 9. signal transducer activity, 10. enzyme regulator activity, 11. antioxidant activity, 12. translation regulator activity, 13. transcription regulator activity, 14. structural molecule activity, 15. transporter activity, 16. molecular function unknown, 17. binding, 18. catalytic activity. Z axis: percentage of ESTs with known GO molecular function in each library, calculated as number of ESTs with GO assignments/total number of EST in each library.

Hot dry summers limit the production of cool season forage and turf grasses including tall fescue, therefore, abiotic stresses constitute challenges to forage production. Heat and drought stress typically overlap during summer tall fescue production, and both stresses may induce plant responses leading to similar physiological changes. To identify genes induced under stress conditions, ESTs were generated from four cDNA libraries constructed from drought stressed shoot (DS1), drought stressed root (DR1), heat stressed shoot (HSS), and field stressed shoot (FSS) tissues. ESTs were assigned GO molecular function annotations, and EST percentages were calculated based on comparison to libraries representing non-stressed, greenhouse-grown tissues to identify stressed-associated genes (Figure 4).

The field-stressed shoot library was constructed using the entire shoot and leaf tissues harvested from field-grown plants during mid-July with high daily average air temperatures ranging from 35.2°C to 38.7°C. Plants were under environmental stresses including heat, drought as well as possible field pathogens, representing severe summer pasture growth conditions in the U.S. southern Great Plains. FSS ESTs were compared to the greenhouse-grown leaf library to examine stress-response of above ground tissues. Many ESTs identified in the field-stressed library include a number of stress-related gene homologoues (e.g., cysteine proteinase, cytochrome P450, and proline-rich proteins). In addition, the percentage of ESTs involved in enzyme regulator activity, based on ontology, was much higher than those from non-stressed leaf (0.74% *vs*. 0.27%) (Figure 4). However, a higher percentage of ESTs having transporter, binding, and catalytic activity GO function may suggest active growth of non-stressed leaf tissue. Large numbers of genes involved in photosynthesis and carbon fixation were found only in the greenhouse-grown leaf library.

Comparison of drought stressed (DR1) *vs*. non-stressed, greenhouse-grown root libraries revealed an elevation in transcription regulator ESTs in drought stressed roots (Figure 4). ESTs of genes coding for components of signal transduction pathways (MAP kinase MAPK2, Ras-related GTP-binding protein), transcription factors (Zinc-finger protein, WRKY transcription factors, and MYB factor), and hormone-mediated signalling proteins (auxin response factor 1, and 2) are included in this group. Homologues of the rice s-adenosylmethionine synthetase gene were highly induced in drought-stressed roots (70 ESTs), while only four ESTs of this gene were found in non-stressed root tissues. Ontology analyses indicate that antioxidant, translation regulator, transporter, binding, and catalytic activities of drought stressed root were suppressed in comparison to greenhouse grown roots (Figure 4). The expression of root-specific metallothionein-like protein genes were significantly decreased in drought-stressed root tissues (0.46%) when compared to roots from greenhouse-grown plants (1.49%). This may suggest physiological processes, including chelation of toxic metal ions and protection against oxidative damage during the process of cellular death and degradation [12], may have been reduced in stressed roots. ESTs unique to drought-stress were also identified. A drought-specific gene (TC1995), which was highly expressed only in drought-stressed root and shoot libraries (928 ESTs – cluster H) showed no similarity to sequences in nr. Between library EST comparisons were made to examine changes in shoot tissue ESTs from tall fescue plants subjected to drought-, heat-, or field-stress (a combination of drought, heat and other environmental stresses). Under multiple stress conditions, field stressed shoots expressed fewer classes of genes compared to heat- (HSS) or drought- (DS1) stress alone (Figure 4). However, many ESTs expressed in at least two of the three libraries were found. Proline-rich protein ESTs were expressed in both DS1 and FSS libraries, and may suggest a common mechanism of producing osmoprotectants in response to drought under either condition. Among the transcripts expressed in field- and heat-stressed shoots, transcripts encoding mitochondrial proteins such as various subunits of NADH dehydrogenase and cytochrome c oxidase were identified. Expression of these transcripts correlated with the enhanced respiratory activity [16] detected in plants subjected to heat stress or field stress. As expected, large numbers of HSPs genes were expressed under both environments. Transcripts expressed under all three treatments include genes for transcriptional factors, oxidative burst, and stress-related homologues (e.g., zinc-finger protein, ubiquitin conjugating enzyme, oxidase, DnaK-type molecular chaperone, wound-responsive family protein, proline-rich protein, jasmonate-induced protein, cysteine proteinase inhibitor, and catalase). Our results indicate universal expression of these genes in response to stress conditions, similar to those reported in other studies [16-19].

### Heat shock proteins and beyond

Due to the important roles of heat shock proteins (HSPs) in multi-stress conditions, we compared the expression of HSPs in all nine cDNA libraries (Table 3). A total 53 HSP gene homologues were identified, representing four of the five classes of heat shock proteins [20]. Based on their predicted molecular mass, tall fescue HSPs were grouped into low molecular weight (15–30 kDa), HSP70 (69–71 kDa), high molecular weight (80–114 kDa), and unclassified HSPs (Table 3), each contains 22, 7, 9, and 15 HSPs, respectively. Although special focus was placed on stress treated tissues, HSPs ESTs were also present in rapidly developing tissues of floral meristems, roots, and young seedlings.

**Table 3:** Tall fescue HSP and heat shock transcription factor (HSF) gene expression

**Unigene ID**^a^	**DR1**	**DS1**	**FSS**	**TFM**	**HSS**	**LF1**	**RT1**	**ST1**	**SD1**	**Annotation**
										**Low molecular weight (LMW) HSPs**
										
TC3677	0 ^b^	0	0	0	4	0	0	0	0	UP|HSP11_WHEAT (P12810) 16.9 kDa class I hsp
DT693759	0	0	0	0	2	0	0	0	0	PRF|1908439B|445140|1908439B hsp 16.9B. {*O. sativa*}
DT696482	0	0	0	0	0	2	0	0	0	UP|Q40866_PENAM (Q40866) hsp 17.0
TC739	0	0	0	0	8	0	0	0	0	UP|Q96458_HORVU (Q96458) 17 kDa class I small hsp
DT706802	0	0	0	0	0	0	0	2	0	PRF|NP_175807.1|17.4 kDa class III hsp {*A.thaliana*}
TC4040	48	0	0	0	2	0	0	0	0	UP|Q94KM0_WHEAT (Q94KM0) Small hsp HSP17.8
TC2206	0	0	0	0	2	0	2	2	0	UP|Q40867_PENAM (Q40867) hsp 17.9
TC2205	0	0	8	0	12	0	0	0	0	UP|Q8W007_ORYSA (Q8W007) Class I lmw-hsp 17.9
DT684410	0	0	8	0	0	0	0	0	0	UP|Q5R1P5_BOMMO (Q5R1P5) hsp21.4
TC2142	0	0	0	0	4	0	0	0	0	UP|O64960_MAIZE (O64960) lmw-hsp (HSP22)
TC1295	0	0	0	0	4	0	0	0	0	PIR|S65051|S65051 lmw-hsp (Hsp22.5) {*G. max*}
TC2141	0	0	0	0	28	0	0	0	0	UP|Q9ZP25_WHEAT (Q9ZP25) sm Hsp23.5 precursor
DT695134	0	0	0	0	2	0	0	0	0	UP|Q41568_WHEAT (Q41568)hsp 26.6B
TC2085	0	0	8	0	18	0	0	0	0	UP|Q8GV35_9POAL (Q8GV35) Chl lmw-hsp HSP26.7b
DT685446	0	0	8	0	0	0	0	0	0	UP|Q8GV37_9POAL (Q8GV37) Chl lmw-hsp HSP26.8
DT693763	0	0	0	0	2	0	0	0	0	UP|O80432_LYCES (O80432) Mitochondrial smhsp
TC4358	0	0	0	0	10	0	0	2	0	PDB|1GME_A|1GME_Eukaryotic smhsp {*T. aestivum*}
TC501	0	0	0	0	12	0	0	0	0	UP|Q40978_PAPSO (Q40978) lmw-hsp
DT695083	0	0	0	0	2	0	0	0	0	UP|Q41218_SOLTU (Q41218) smhsp homolog
DT684541	0	0	8	0	0	0	0	0	0	UP|Q4ZHH0_9BASI (Q4ZHH0) smhsp frag
DT713365	0	0	0	0	0	0	0	0	2	UP|Q58FS1_TRIHA (Q58FS1) smhsp

**Unigene ID**	**DR1**	**DS1**	**FSS**	**TFM**	**HSS**	**LF1**	**RT1**	**ST1**	**SD1**	**Annotation**

TC4794	0	0	0	0	4	0	0	0	0	UP|Q8L470_LYCES (Q8L470) smhsp
										**HSP 70**
										
DT679789	0	2	0	0	0	0	0	0	0	GB|CAA54419.1|450880|ATHSC701 hs cognate 70-1 {*A. thaliana*}
TC64	0	0	0	0	2	0	0	4	0	UP|HSP72_LYCES (P27322) hs cognate 70 kDa prot 2
DT691644	0	0	0	0	2	0	0	0	0	UP|HSP74_HUMAN (P34932) hs 70 kDa protein 4 (HSP70RY)
DT695482	0	0	0	0	2	0	0	0	0	UP|HSP7S_PEA (Q02028) Stromal 70 kDa hs-related protein
DT682340	0	2	0	0	0	0	0	0	0	UP|O50036_SPIOL (O50036) hs 70 protein
DT692072	0	0	0	0	2	0	0	0	0	UP|O04056_CITLA (O04056) hsp precursor
TC63	8	14	0	38	4	4	21	2	0	UP|Q84TA1_ORYSA (Q84TA1) hspgnate 70

										**High molecular weight HSPs**
										
DT692018	0	0	0	0	2	0	0	0	0	UP|Q9ZRG0_WHEAT (Q9ZRG0) hsp
TC149	0	0	0	4	2	4	2	2	0	UP|HSP82_ORYSA (P33126) hsp
TC5768	0	0	0	0	4	0	0	0	0	PIR|A48426|A48426 HSP82 – maize {*Z. mays*}
TC148	0	0	0	12	2	0	4	10	4	UP|Q7XJ80_HORVU (Q7XJ80) Cytosolic hsp 90
DT684503	0	0	8	0	0	0	0	0	0	UP|Q45XA1_BEMTA (Q45XA1) 90 kDa hsp
DT710057	0	0	0	0	0	0	0	2	0	UP|HS101_ORYSA (Q6F2Y7) hsp 101
DT697638	0	0	0	0	0	2	0	0	0	UP|Q6Z517_ORYSA 101 kDa hsp; HSP101-like
TC2500	0	0	0	0	2	0	0	0	8	UP|Q9LF37_ARATH (Q9LF37) ClpB hsp -like
TC950	0	0	0	2	0	0	0	0	4	UP|HS105_HUMAN (Q92598) hsp 105 kDa

										**Unclassified HSPs**
										
TC5675	0	0	0	0	0	0	0	0	4	UP|O23638_ARATH (O23638) hsp precursor
DT708187	0	0	0	0	0	0	0	2	0	UP|O49457_ARATH (O49457) hsp
TC3195	0	0	0	0	2	0	2	0	0	UP|P93437_ORYSA (P93437) hsp
TC2515	0	4	0	0	0	2	0	0	4	UP|Q43638_SECCE (Q43638) hsp
TC1929	0	0	0	0	4	0	0	0	0	UP|Q4LDR0_LYCES (Q4LDR0) hsp

**Unigene ID**	**DR1**	**DS1**	**FSS**	**TFM**	**HSS**	**LF1**	**RT1**	**ST1**	**SD1**	**Annotation**

TC4728	0	0	8	0	4	0	0	0	0	UP|Q5ZBN6_ORYSA (Q5ZBN6) hs-like protein
DT702768	0	0	0	0	0	0	2	0	0	UP|Q6ER93_ORYSA (Q6ER93) hsp -like
TC1335	0	0	0	2	0	0	2	0	0	UP|Q6K2F0_ORYSA (Q6K2F0) hsp -like
TC3822	0	0	0	0	0	0	2	2	0	UP|Q96269_ARATH (Q96269) hsp
TC3007	0	0	0	0	0	0	2	0	4	UP|Q9FHQ0_ARATH (Q9FHQ0) Calmodulin-binding hsp
DT710639	0	0	0	0	0	0	0	2	0	UP|STIP_SOYBN (Q43468) hsp STI (Stress inducible pron) (GmSTI)
DT710259	0	0	0	0	0	0	0	2	0	PRF|NP_175842.1 hs family protein {*A. thaliana*}
DT690417	0	0	0	2	0	0	0	0	0	PRF|NP_187434.1|NP_187434 hsp -related {*A. thaliana*}
TC4128	0	4	0	4	4	0	9	4	0	PRF|NP_191819.1 DNAJ hs family protein {*A. thaliana*}
DT679933	0	2	0	0	0	0	0	0	0	PRF|NP_194764.1 hsp -related {*A. thaliana*}

										**HSFs**
										
DT694163	0	0	0	0	2	0	0	0	0	UP|Q6VBB5_ORYSA (Q6VBB5) hsf RHSF2
DT694677	0	0	0	0	2	0	0	0	0	GB|AAQ23057.1|33591100|hsf RHSF3 {*O. sativa*}
DT688511	0	0	0	2	0	0	0	0	0	GB|AAQ23059.1|33591104|hsf RHSF5 {*O. sativa*}
TC2134	14	0	0	0	10	0	0	0	8	GB|AAQ23060.1|33591106 hsf RHSF6 {*O. sativa*}
TC5588	0	0	0	0	4	0	0	0	0	GB|AAQ23061.1|33591108|heat shock factor RHSF7 {*O. sativa*}
DT705016	0	0	0	0	0	0	2	0	0	GB|AAQ23062.1|33591110|hsf RHSF8 {*O. sativa*}
TC3473	0	0	0	0	4	0	0	0	0	UP|Q657C0_ORYSA (Q657C0) hs tf HSF8-like
DT679516	0	2	0	0	0	0	0	0	0	UP|Q6Z7B3_ORYSA (Q6Z7B3) hsf protein hsf8-like
TC3759	0	0	0	0	0	0	4	0	0	UP|Q94J16_ORYSA (Q94J16) hsf RHSF9
TC1477	0	2	0	0	2	0	0	0	0	UP|Q5Z9D6_ORYSA (Q5Z9D6) hsf RHSF13-like

^a^Unigene ID. DT = singleton, TC = tentative consensus, Drought stressed root = DR1, Drought stressed shoot = DS1, Field stressed shoot = FSS, Floral meristem = TFM, Heat stressed shoot = HSS, Greenhouse grown leaf = LF1, Greenhouse grown root = RT1, Field grown stem = ST1, Young seedling = SD1.

^b ^Numbers of normalized ESTs found matching known HSPs or HSFs were listed in individual library.

Generation of low molecular weight (LMW) or small (sm) heat shock proteins is one of the unique aspects of the heat shock response in plants [21]. Six classes of smHSPs have been identified in stressed plants [22]. Although the function of smHSPs has not been defined, evidence suggests that they serve as molecular chaperones to protect cells from stress damage, but that they are not required for normal cellular function [23]. Our EST analysis showed that 16 LMW-HSP genes were expressed in the heat stressed shoot library, which is significantly different from their expression in other libraries (Table 3). The expression of five LMW-HSP genes in field-stressed shoot tissues may reflect the high temperatures encountered during summer. Previously we found that LMW-HSP genes were highly induced in heat treated tall fescue but not detectable in non-stressed plants [24]. Sun *et al*. [25] found the expression of *HSP17.6A*, a cytoplasmic class II smHSP gene in *Arabidopsis *was triggered by changed water potential and was critical in osmotic stress tolerance. TC4040 codes for a wheat smHSP17.8 homologue that was highly expressed in drought-stressed roots (48 ESTs), however, no expression was detected in drought-stressed shoot.

HSP70 proteins have been proven to be essential for normal cell function [26]. Some members of the HSP70 family are expressed constitutively while others are induced by heat or cold [27]. TC63 was expressed in seven of the nine libraries (Table 3). Interestingly, more than twenty ESTs of this TC were found in floral meristem and greenhouse grown root libraries- tissues active in cell division and elongation.

Divergent from LMW-HSP and HSP70 gene transcripts, expression of high molecular weight HSP genes occurred mostly in developing tissues (floral meristem, greenhouse grown root, stem, and young seedlings) rather than in stressed tissues. For example, TC148, which encodes a barley cytosolic HSP90 homologue, was represented by multiple ESTs in all developing tissue libraries. As a chaperone complex in plants, HSP90 functions in response to external stimuli (abiotic stresses and pathogens) and it is involved in phenotypic plasticity, developmental stability, and buffering of genetic variation [28]. A gene homologue of ClpB, a subfamily of HSP100 [29], had mid-level expression in young seedling tissues in addition to expression in heat-stressed shoots of tall fescue. A previous study has shown this nuclear-localized protein has a negative influence to the growth rate of the primary root in addition to its role in thermotolerance in maize [30].

Because the expression of HSPs is regulated by the activity of heat shock transcription factors (HSFs) [31], the expression pattern of HSF genes was examined for tall fescue. We identified ten HSF genes representing eight classes of HSFs, all of which had homologues in *O. sativa*. It is well accepted that at least two families of heat shock transcription factors (HSFs) exist in plants: Class A HSFs are primarily responsible for stress-inducible activation of heat shock genes whereas some of the class B HSFs may be specialized for repression, or down-regulation, of the heat shock response [32]. This may explain the difference of the expression patterns for ten tall fescue HSF genes. For example, DT694677 which codes for HSF3 homologue was expressed in heat-stressed shoot tissue only. This is similar to findings regarding AtHSF3, which functions as a key regulator of the immediate stress-induced activation of heat shock gene transcription in *Arabidopsis *[33]. A HSF6 gene homologue (TC2134) had high levels of expression in drought-stressed roots, heat-stressed shoots, and young seedlings, and therefore, may function in both stressed and developing tissues. HSFA9 is a transcription factor critically involved in developmental activation of hsp17.6G1 and specifically expressed during embryogenesis in sunflower in the absence of environmental stress [34]. A tall fescue homologue of RHSF9 was expressed in greenhouse grown root tissues.

Expression of ascorbate peroxidase (APX), a defence enzyme controlled by HSFs and elevated during heat stress was also examined [35]. APX isoenzymes are critical components that prevent oxidative stress in photosynthetic organisms by removing reactive oxygen intermediates [36]. Six genes coding for APX homologues were identified in our tall fescue EST gene index, all of which were expressed in heat stressed shoot. Expression of these genes was also present in eight of the nine cDNA libraries, but not in field stressed shoot library (data not shown). A previous study has shown that the expression of APX was induced shortly after tall fescue plants were exposed to heat (12 hrs at 39°C) [37]. However, APX expression was reduced with increased temperatures and exposure time. Therefore, it is not surprising to find low (or no) expression of APX in plants grown under prolonged high-stress (field) conditions.

## Conclusion

Prior to this study, little or no genomic information was available for tall fescue. In this study, we analyzed 41,516 tall fescue ESTs from nine cDNA libraries representing different abiotic stresses, tissue types and developmental stages. A large number of known stress response gene ESTs were identified from stressed tissue libraries. These ESTs represent gene homologues of heat-shock and oxidative stress proteins, and various transcription factor protein families. The EST database reported herein has been used for the development of 145 EST-SSR markers [38] that have been used to generate a genetic linkage map of *Festuca *[39]. The development of additional EST-SSR markers is underway [40]. A study using tall fescue EST-SSR markers in a phylogenetic analysis of 12 cool-season forage grass species representing eight genera of four tribes from two subfamilies of *Poaceae *demonstrated the utility of these markers for comparative genomics studies among grass species [41].

The EST characterized in this study can be used for the development of microarrays and mined for single nucleotide polymorphism markers. This database provides a basis for further gene discovery and the determination of gene function. Additional cDNA libraries representing tissues subjected to other abiotic stress factors, e.g., phosphate starvation, high salt stress, summer field stress, not included in this study will enrich this database. Moreover, an important growth component of tall fescue, endophyte-host interactions, has not been studied in relation to gene expression. Development of ESTs from fungal endophyte-infected tall fescue tissue may assist in better understanding of plant-fungal symbiotic relationships.

## Methods

### cDNA library construction

Nine cDNA libraries were constructed to characterize developmental stage-, environmental stress-, and tissue-specific ESTs (Table 1). Details on the libraries are available from GenBank dbEST [42]. Endophyte-free tall fescue (cv. Kentucky 31) plants were used for cDNA library construction. In an out-crossing species, plants within a cultivar (population) are not genetically identical. Therefore, tissues were collected from 25 plants at each sampling time point in order to account for the genetic variation within the cultivar. Total RNA was extracted by Tri-Reagent (MRC, Cincinnati, OH). Quality and quantity of RNA were assessed using both an Eppendorf Biophotometer (Eppendorf, Hamburg, Germany) and formamide gel electrophoresis according to standard protocols. An equal amount of each appropriate RNA sample was pooled together to form a composite total RNA sample. mRNA isolation to construct the cDNA library. The mRNA was purified by Oligotex mRNA Midi kit (Qiagen, Valencia, CA). cDNA libraries were constructed using the ZAP-cDNA Gigapack III Gold Cloning Kit (Stratagene, La Jolla, CA). cDNA was size fractionated following manufacturers instructions. Fractions containing cDNA greater than 1,000 bp were selected for library construction.

### DNA sequencing and data processing

Plasmid isolation and DNA sequence analyses were performed on an ABI 3730 DNA Analyzer using standard procedures. Sequence data generated from this project were processed through ESTAP [43]. Sequences were screened for overall base quality and contaminating vector, mitochondrial, ribosomal and *E. coli *sequences were removed.

### EST assembly and annotation

Sequences with either short length (<100 bp) or low complexity were excluded from the gene index. High quality ESTs were clustered and assembled by TGI Clustering tools (TGICL) [44]. Tentative consensus (TC) and singleton EST sequences are available for downloading from The Dana Faber Cancer Center [45]. A batch BLASTX [46] comparison of the ESTs to the GenBank nr database [47] was performed. A BLASTP search was performed against the database of sequences associated with GO gene products which was downloaded from the GO website [48] along with GO controlled vocabulary terms and their relationships. Following annotation, a Perl script embedded with SQL statements was executed to count the number of members for each library at different go term branch and sub-branches.

### In silico analysis of gene expression

Based on the assumption that the occurrence of a gene in a cDNA library may represent the level of its tissue-specific expression, TC member numbers were counted according to the corresponding library by Perl and Unix bash scripts, and a matrix exhibiting the expression differentiation of TCs across the nine libraries was generated. In this matrix, TCs and their membership size in each library are provided in each row and column, respectively. The data matrix was normalized to compensate for variation in EST sample size among libraries. Normalized gene expression level in each library was calculated as: TC membership size in a library/total number of ESTs of the library × 10,000. Data was imported into GeneSpring GX 7.3 (Agilent Technologies, Palo Alto, CA) for expression analysis. To compare between libraries, hierarchical clustering analysis was performed on the matrix of *in silico *gene expression pattern (counts of normalized EST copy numbers) generated by tracking unigene membership from all nine libraries. The clustering algorithm implemented by the GeneSpring software package measures similarity of *in silico *gene expression pattern by calculating "standard correlation" and further generates hierarchical clusters using the "average linkage" clustering algorithm. Figure 3 shows a heat map of the data matrix with data organized based on gene tree and condition tree clusters.

## Authors' contributions

YZ, JZ, XC, LC, MARM constructed cDNA libraries; KC, ADS conducted sequencing; YZ, MARM, ZW, GDM contributed to the manuscript writing; XD, JH, XZ, CM conducted the bioinformatics; FC, CDT established TIGR FaGI; MARM and GDM managed the overall project. All authors read and approved the final manuscript.
